# Redefining Genomic Privacy: Trust and Empowerment

**DOI:** 10.1371/journal.pbio.1001983

**Published:** 2014-11-04

**Authors:** Yaniv Erlich, James B. Williams, David Glazer, Kenneth Yocum, Nita Farahany, Maynard Olson, Arvind Narayanan, Lincoln D. Stein, Jan A. Witkowski, Robert C. Kain

**Affiliations:** 1 Whitehead Institute for Biomedical Research, Nine Cambridge Center, Cambridge, Massachusetts, United States of America; 2 Google Inc., Mountain View, California, United States of America; 3 Illumina Inc., San Diego, California, United States of America; 4 Duke University School of Law, Duke Science & Society, Durham, North Carolina, United States of America; 5 University of Washington, Port Orford, Oregon, United States of America; 6 Department of Computer Science, Princeton University, Princeton, New Jersey, United States of America; 7 Ontario Institute for Cancer Research, Toronto, Ontario, Canada; 8 Department of Molecular Genetics, University of Toronto, Toronto, Ontario, Canada; 9 Banbury Center, Cold Spring Harbor Laboratory, Huntington, New York, United States of America; King's College London, United Kingdom

## Abstract

Fulfilling the promise of the genetic revolution requires the analysis of large datasets containing information from thousands to millions of participants. However, sharing human genomic data requires protecting subjects from potential harm. Current models rely on de-identification techniques in which privacy versus data utility becomes a zero-sum game. Instead, we propose the use of trust-enabling techniques to create a solution in which researchers and participants both win. To do so we introduce three principles that facilitate trust in genetic research and outline one possible framework built upon those principles. Our hope is that such trust-centric frameworks provide a sustainable solution that reconciles genetic privacy with data sharing and facilitates genetic research.

## Abstract 2

Current models of protecting human subjects create a zero-sum game of privacy versus data utility. We propose shifting the paradigm to techniques that facilitate trust between researchers and participants.

## Introduction: The Rise and Fall of De-identification

*“Widespread distrust…imposes a kind of tax on all forms of economic activity, a tax that high-trust societies do not have to pay.”–Francis Fukuyama* [1]

Genomic research promises substantial societal benefits, including improving health care as well as our understanding of human biology, behavior, and history. To deliver on this promise, the research and medical communities require the active participation of a large number of human volunteers as well as the broad dissemination of genetic datasets. However, there are serious concerns about potential abuses of genomic information, such as racial discrimination and denial of services because of genetic predispositions, or the disclosure of intimate familial relationships such as nonpaternity events. Contemporary data-management discussions largely frame the value of data versus the risks to participants as a zero-sum game, in which one player's gain is another's loss [2],[3]. Instead, this manuscript proposes a trust-based framework that will allow both participants and researchers to benefit from data sharing.

Current models for protecting participant data in genetic studies focus on concealing the participants' identities. This focus is codified in the legal and ethical frameworks that govern research activities in most countries. Most data protection regimes were designed to allow the free flow of de-identified data while restricting the flow of personal information. For instance, both the Health Insurance Portability and Accountability Act (HIPAA) [4] and the European Union privacy directive [5] require either explicit subject consent or proof of minimized risk of re-identification before data dissemination. In Canada, the test for whether there is a risk of identification involves ascertaining whether there is a “serious possibility that an individual could be identified through the use of that information, alone or in combination with other available information” [6]. To that end, the research community employs a fragmented system to enforce privacy that includes institutional review boards (IRBs), ad hoc data access committees (DACs), and a range of privacy and security practices such as the HIPAA Safe Harbor [7].

The current approach of concealing identities while relying on standard data security controls suffers from several critical shortcomings (Box 1). First, standard data security controls are necessary but not sufficient for genetic data. For instance, access control and encryption can ensure the security of information at rest in the same fashion as for other sensitive (e.g., financial) information, protecting against outsiders or unauthorized users gaining access to data. However, there is also a need to prevent misuse of data by a “legitimate” data recipient. Second, recent advances in re-identification attacks, specifically against genetic information, reduce the utility of de-identification techniques [8],[9]. Third, de-identification does not provide individuals with control over data—a core element of information privacy [10].

**Box 1. The Gaps in Current Data Privacy Techniques**

It may be that current technological methods for privacy protection, which primarily consist of removing an individual's personally identifying information from records containing individualized genetic information, are simply outdated; it is possible that new techniques will once more make it difficult to infer personal information. Here, we briefly review computational schemes that theoretically make re-identification demonstrably (and perhaps quantifiably) difficult. For a comprehensive technical overview, please refer to [27].

In general, there are two classes of advanced privacy-preserving techniques relevant to genetic data: cryptographic techniques and statistical techniques. The hallmark of all of these techniques is that they provide mathematical proofs delineating what the data recipient can and cannot infer based on the data access given to them.

Cryptographic techniques can compute a known, shared function on encrypted datasets from multiple parties; the computation reveals nothing about the parties' input data other than the function's results. For example, a patient or her physician holding genetic data can use such a technique to have the genetic data interpreted by a third-party service for disease susceptibility without revealing the actual genotypes. However, cryptographic techniques have some practical limitations. For instance, they require predefined analysis protocols. Research protocols are rarely fixed in advance. Most research is exploratory in nature and is characterized by ad hoc analyses in which researchers test and refine their analytic procedures repeatedly during the course of the study. Moreover, the final output of cryptographic techniques has to be decrypted to be useful. Thus, while these techniques enable secure computation of the raw data, the final product is still vulnerable to certain attacks and its broad dissemination can create privacy concerns.

Statistical techniques work by adding noise to the disseminated data. The premise of these methods is that in some scenarios the amount of noise needed to conceal the identity of individuals in the dataset is quite small and still permits accurate detection of general phenomena in the data. Unfortunately, in genomics, the current levels of noise required to reduce privacy risks appear to be unacceptable because of the richness of the information and the uniqueness of one's genome. Empirical tests showed that these techniques can eradicate the weak association signals that are the reality of most complex traits.

Our conclusion is that these emerging computational techniques for ensuring genetic privacy show potential but would require substantial theoretical and practical development to be fully operational methods for data sharing to accelerate scientific studies.

With the growing limitations of de-identification, the current paradigm is not sustainable. At best, participants go through a lengthy, cumbersome, and poorly understood consent process that tries to predict worst-case future harm. At worst, they receive empty promises of anonymity. Data custodians must keep maneuvering between the opposite demands for data utility and privacy, relegating genetic datasets into silos with arbitrary access rules. Funding agencies waste resources funding studies whose datasets cannot be reused across and between large patient communities because of privacy concerns. Finally, well-intentioned researchers struggle to obtain genetic data from hard to access resources. These limitations impede serendipitous and innovative research and degrade a dataset's research value, with published results often overturned because of small sample sizes [11].

## Focusing on Trust Not Privacy

We propose to shift from the zero-sum game of data privacy versus data utility to a framework that builds and maintains trust between participants and researchers. We suggest the following key principles for trust-enabling frameworks:

1. **Transparency creates trust:** Trust requires transparency between parties. In genomic research, transparency means informing participants about not only the intended but also the actual use of data. This is a commonly accepted principle of information privacy that is found in most data protection statutes (e.g., Canada's Personal Information Protection and Electronic Documents Act [PIPEDA] [12]) and fair information practices (e.g., the Organisation for Economic Co-operation and Development [OECD] Privacy Principles [13]).
2. **Increased control enhances trust:** Given the uncertainties in genetic studies, the burden of making “fully informed” decisions about future data use and harms is virtually impossible. However, the situation improves when the participant is given control over future data use. Clear communication of risks is crucial to ensure fully informed participants, yet current consent processes require participants to make a one-time decision about future data sharing preferences with unknown risks. Even worse, some consent forms include vague “legalese” that might be tempting from a legal perspective but instead fuels patients' fears. Some participants naturally shy away from sharing when the terms are too broad, while other individuals might make decisions that are not well informed. In addition, one-time “blanket” consent does not accommodate the reality that privacy preferences might change over time.
3. **Reciprocity maintains trust:** Researchers should maximize the value of data collected from participants, subject to individual preferences. By advancing scientific knowledge, the research community reciprocates and “pays back” the participant's volunteerism. A sense of community among participants can help bridge the gap between societal and individual rewards. Mechanisms for participants to “reward” researchers who act appropriately (and “punish” researchers who violate their trust) provide incentives for ongoing win-win behavior.

If successful, a trust-centric framework creates a system that rewards good behavior, deters malicious behavior, and punishes noncompliance. This stands in stark contrast to the current system that punishes researchers, participants, and progress.

## Bilateral Consent Framework

Building on top of the three key principles above, we suggest a trust-enabling framework, called the Bilateral Consent Framework (BCF) (Table 1). This approach is inspired by the recent movement for participant-centered research [14] and the growing success of online peer-to-peer marketplaces such as Airbnb or Uber that rely on trust-enabling techniques [15]. To be clear, our proposal is not meant to be final but rather to provide a framework and a set of building blocks to drive discussions among the community. The major building blocks of the BCF are introduced in the following subsections.

**Table 1:** Major differences between current data sharing frameworks and a BCF.

Attribute	Current System	BCF
Consent for secondary use	One-time decision	Dynamic
Primary data controller	PI	Participant
Who decides on secondary data usage?	DAC or local IRB	Participant
Data stewardship	Not defined	Trusted mediator
Code of conduct	Locally determined	Globally determined
Oversight	Local IRB	The community (participants, trusted mediator, and researchers)
Oversight mechanism	Not clear	Audit system
Who can punish data misconduct?	Local IRB	The community (participants, trusted mediator, and researchers)
Main source of reputation	University or research institute	The community (previous participants, trusted mediator, and researchers): participant ratings, previous studies, peer researcher recommendations, reputation of host organization, auditing reports, researcher's history of results, etc.
Cohort integrity	Stable	Indefinite/variable
Place of computation	PI-owned equipment or PI-chosen cloud provider	Resource-owned equipment or resource-chosen cloud provider.

## Trusted mediator

The role of the trusted mediator is to operate the BCF. This entity can be any organization that (1) is trusted by the participants and (2) has the means to operate the BCF. It could be a patient advocacy group (e.g., National Breast Cancer Coalition), a funding agency (e.g., National Center for Biotechnology Information [NCBI]), a genome center (e.g., New York Genome Center or the Broad Institute), a scientific society (e.g., American Society of Human Genetics), or a private company (e.g., Illumina or Beijing Genomics Institute [BGI]). It should mediate the communication between the researchers and the participants, act upon the participants' decisions, and be the single point of contact. In addition, this entity should educate participants about the nature of the data and describe the benefits and risks.

## Uniform code of conduct

Having researchers consent to uniform guidelines makes it easier for participants to grant consent to new researchers. Researchers who are part of the BCF consent to a code of conduct that affirms that individual data will be properly handled, including that it will be held securely and that re-identification will not be attempted. Thus, BCF replaces the “gatekeeper” approach, wherein IRBs decide who should count as a qualified researcher on a case-by-case basis, with a participant-centric model, in which participants understand the rules that researchers will follow. Evidence for violation of the code of conduct can result in public notice, canceled access, and possible legal action. Methods for redress might include data protection law, criminal law, or additional contractual terms, such as indemnification and compensation, similar to the model suggested by Prainsack and Buyx [16].

## Auditing

The BCF encourages a “trust-but-verify” approach. All data access should be monitored, both to remind researchers that their access privileges depend on trust and to enable potential detection of violations and enforcement of obligations. One means of monitoring is for all analysis activity to be executed on the trusted mediator's computing resources and logged. This is different from current access control models in which (upon permission) the researcher analyzes the data on his or her own computing resources without any oversight on the actual analysis. Importantly, we do not expect the auditing system to be perfect or to capture all data misuse. The primary aim of such a system is to deter malicious behavior. However, we envision that in the future such systems can help to automatically identify clear anomalies (e.g., the analysis of short tandem repeats on the Y-chromosome [Y-STRs] that is a key component of surname inference [9]) or data analysis that is substantially different from the consent. In addition, logging and auditing promote transparency. There is growing interest in using cloud computing for genetic analysis and moving the computation to the data; adding an auditing system can leverage this trend to increase trust.

## Reputation system

Reputation systems have revolutionized online sharing marketplaces, enabling strangers to trust each other with their safety (e.g., a reckless driver in an Uber car), privacy (e.g., a hidden camera in an Airbnb room), property (e.g., ruining a car in RelayRides), or task integrity (e.g., a lazy worker in Amazon Mechanical Turk). These systems usually consist of an initial background check by the service mediator that grants permission to use the service, followed by ongoing rating of the participants. In some services, such as Uber, when the reputation drops below a certain threshold, the participant is banned from using the service.

Similarly, we propose a reputation system to facilitate researcher good conduct and maintain participant responsiveness. Such a reputation system would reward researchers who maintain solid records of adherence to the code of conduct by elevating their visibility and reputation. The researcher reputation system can incorporate several measures, such as the following: (a) ratings from previous study participants, (b) the number and impact of previously accomplished studies, (c) recommendations from peer researchers, (d) the reputation of the researcher host organization, (e) auditing system reports about the sensitivity of the analysis, and/or (f) the researcher's history of returning results and raw data to participants or publishing previous manuscripts in open-access journals. Accordingly, participants can elect to share data only with researchers of sufficient reputation, and the trusted entity can revoke access to researchers with a low reputation.

The reputation system can also be extended to include the participants. For instance, it could summarize their contribution to studies and overall participation. Similar systems are common in online communities that rely on volunteers, such as Stack Overflow. Empirical research has shown that these systems can create strong incentives for online participants, resulting in increased participation [17]. In the context of the BCF, we believe that such a system can not only increase participation but also foster the development of long-term relationships with participants.

## Dynamic participant consent

At its core, the BCF enables participants to have dynamic control over access to data about them. In current consent architectures, the participant delegates complete control over the data to the principal investigators (PIs). Upon completion of the study, the PI typically delegates secondary usage decisions to a DAC or an IRB. In the BCF, data control remains primarily tied to the source individual. Researchers solicit their studies, describing the benefits of the study and specifying limitations on how they use the data. The participant can grant or deny consent to different studies. Thus, instead of one-time decisions about data sharing, a BCF fosters long-term engagement by participants, allowing researchers to solicit participant data while simultaneously empowering participants to change their data contribution as they see fit.

Previous works (e.g., [18]–[20]) have discussed aspects of dynamic consent, including concerns over the implications of participant withdrawal. Although a full resolution is out of scope for this overview, we believe that many of these difficulties can be overcome with appropriate design. For example, one can attempt to mitigate the impacts of withdrawal by carefully circumscribing at which point a participant may withdraw consent. In order to reduce the burden on participants, the system could provide personalized opt-out/opt-in preferences that would automatically accept a study request based on the subject of the study and reputation of the researcher. The participant would receive a periodic digest (e.g., weekly email) of studies that meet her personalized criteria, and if she did not opt out within a certain time frame, her data would be included. The trusted mediator could ask participants to actively review and renew their preferences every few months and disable accounts that did not do so.

We are not alone in our advocacy of dynamic consent. Active research on this topic is underway (e.g., [21],[22]), and commercial offerings like PatientsLikeMe and 23andMe are currently using dynamic consent models [23]. The BCF's dynamic consent mechanism emphasizes reciprocity (also discussed in [14]) and agency, giving participants greater information on researchers and their studies. It envisions data sharing and consent as a shared process (e.g., [24]) involving iteration and feedback.

## The Path Forward

The description above describes core architectural elements of a trust-centric framework. While these building blocks reinforce each other, they are not meant to be an all-or-nothing monolithic system. Implementations of the BCF framework in specific contexts require decision makers to make different choices about which elements to include as well as the fine-grain details of how to include them. For example, the reputation and dynamic consent systems will need to be tuned to maintain participant responsiveness for study durations and to avoid data withdrawal from the later stages of a study. The consent mechanism and language will still need to accommodate and comply with current regulatory schemes, and the reputation system will need to be tuned to avoid reputation bias (e.g., against early-stage investigators).

## Conclusion

Realizing a bilateral consent framework will require new technologies and hard choices. However, there is a need for improved global standards for legal and technical frameworks to share genomic data. Initiatives such as the Global Alliance for Genomics and Health [25] and the Genetic Alliance [26] have started the dialogue; it is our hope that the proposed framework can act as a starting point as stakeholders move from discussion to practice. A bilateral consent framework can transform fears of unknown privacy abuse into excitement for participating in the genetic information revolution.
